# The experimental procedures of sand and lime as base soil stabilization materials on the modified proctor values for flexible pavement construction

**DOI:** 10.1016/j.mex.2023.102473

**Published:** 2023-11-03

**Authors:** Dandung Novianto, Taufiq Rochman

**Affiliations:** a Senior Lecturer of Civil Engineering Dept., State Polytechnic of Malang, Jl. Soekarno Hatta no. 9, Malang 65141, Indonesia; b Professor of Structural Engineering, Civil Engineering Dept., State Polytechnic of Malang, Jl. Soekarno Hatta no. 9, Malang 65141, Indonesia

**Keywords:** Stabilization, Sand, Lime, Atterberg limit, Modified compaction, Optimum moisture content

## Abstract

During road construction, one of the major challenges encountered is dealing with weak subgrade soil, specifically expansive soil that experiences volume changes due to variations in moisture content. Lime stabilization is a widely used method for improving post-construction stability, offering cost savings, and reducing environmental impact. This study reviews various methods for creating soil-lime mixtures, comparing testing methods. Effective soil stabilization leads to improved construction outcomes, cost reduction, and minimized environmental impact. Soil characteristics, including Maximum Dry Density (MDD) and Optimum Moisture Content (OMC) value, are important factors in determining suitability for construction. Therefore, this study focused on soil characterization, grain analysis, Atterberg limits, and modified compaction before and after lime as well as sand stabilization. This study also determined soil characteristics, grain gradation, Atterberg limit, and modified compaction, and also developed implementation methods and budget plans for stabilized earthworks. Subsequently, the tests included moisture content, density, specific gravity (Gs), sieve analysis, grain analysis with a hydrometer, Atterberg limits, and modified compaction. The tests were conducted with varying percentages of sand (20 %) and lime (5 %, 10 %, 15 %, and 20 % of soil dry weight). Original soil moisture content value (w) = 53.70 %, Wet soil unit weight (γ) = 1.69 gr/cm^3^, Gs = 2.69, The original soil is classified into A-2–7 as (silty gravel or clay and sand) according to AASHTO.

• This method shows the effect of sand and lime as base stabilization materials.
• This method examines the roles of modified proctor on the flexible pavement.
• This study explores MDD and OMC for curing times of 1, 7, and 14 days.

## Graphical abstract

**Figure:**
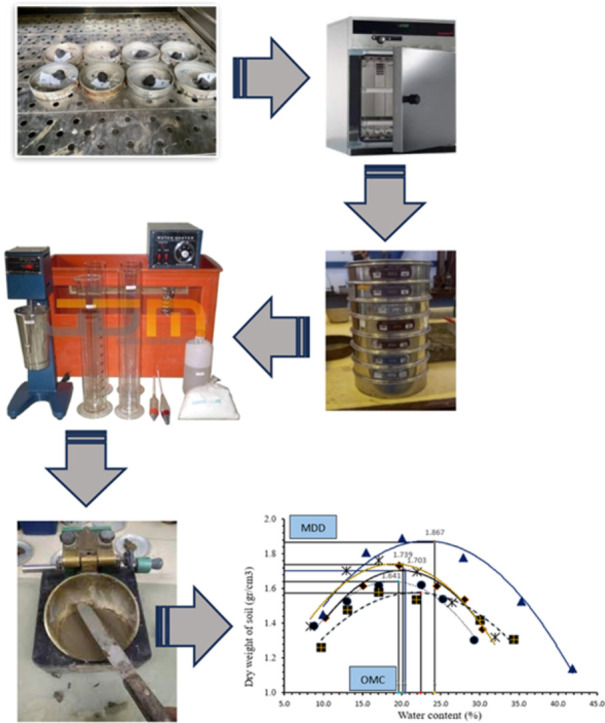

Specification table

**Table:** 

Subject Area:	Civil Engineering
More specific subject area:	Geotechnics for pavement
Method name:	Optimum moisture content
Name and reference of original method:	Rimbarngaye, A., Mwero, J. N., & Ronoh, E. K. (2022). Effect of gum Arabic content on maximum dry density and optimum moisture content of laterite soil. *Heliyon*, *8*(11).
Resource availability:	N/A

**Method Detail**

## Background

In this study, the filling materials used possess a high level of strength and adhere to the necessary gradation standards, with laboratory analysis being crucial in order to meet the necessary regulatory standards. To determine the optimal moisture content and maximum dry unit weight, Proctor tests, a well-established method used for nearly a century was used [1]. The durability of the construction built on soil depends on soil strength, and the compaction characteristics were evaluated through the implementation of modified Proctor compaction tests. Additionally, the strength characteristics were investigated by conducting tests to determine the unconfined compressive strength (UCS) and split tensile strength (STS) [2]. According to the investigation by [3], it is a common practice to re-compact a singular batch sample in order to establish Proctor curves. The acceptance of procedural differences among various Proctor standards worldwide relies on the expectation that the resulting outcomes will show a substantial degree of similarity if not complete equivalence. In line with this investigation, it was stated that the acceptance of procedural variations according to Proctor compaction standards led to minimal variations in moisture content, which have the potential to cause significant fluctuations in the maximum dry density [3]. Another study by [4] examined compacted soils at various moisture contents along the Proctor curve, and the porosity was determined using Mercury Intrusion Porosimetry (MIP) method. Additionally, the study by [5] investigated the influence of various sizes and shapes of rubber on the Proctor characteristics of the CBC mixture. Subsequently, it was noted that the rubberized mixtures showed lower maximum dry density (MDD) values in comparison to the reference mixture. According to the study, it was reported that the optimum moisture content (OMC) of rubberized mixtures shows a decrease in comparison to the sand mixture, while it shows an increase with an increase in the size of rubber particles [5].

A. Proctor compaction method

In order to investigate the particle packing properties that maximize the inter-particle contact of coarse aggregate particles, various laboratory compaction methods were used [6]. These methods included: (1) the “non-compaction” method; (2) the “manually dry-rodded” method; (3) Proctor compaction using a standard rammer; (4) Proctor compaction using a specifically designed rammer; and (5) compaction using a steel roller compactor. The results showed that using new laboratory compaction methods, as detailed in [6], such as the Proctor and steel roller compactor, leads to particle packing that closely resembles field conditions when compared to the other methods studied [6]. However, as discussed by [7], it is important to acknowledge the limitations of the Proctor compatibility test, as it does not consider the potential impact of soil aggregate stability, porosity, and other soil structural properties on the process of soil compaction in real-world field conditions [7]. Subsequently, soil with and without lime additives show characteristic compaction curves as observed in the Proctor test. The optimal moisture content shows a negative correlation with the increase in compaction power, while the maximum dry density shows an inverse relationship. an optimal quality can be achieved by using a moderate level of compaction power [8].

Despite the emergence of new soil compaction technologies, such as intelligent compaction, it was reported that the Proctor test for laboratory soil compaction has remained unchanged for several decades. In addition to determining MDD and OMC, laboratory impact compaction provides additional parameters such as the locking point, compaction curve, and target stiffness of soil, and these parameters can be used to guide the process of field compaction [9]. The use of both the normal Proctor and kneading compaction modes facilitates the achievement of samples with a greater degree of homogeneity in the structure, preventing the formation of preferential failure paths. Therefore, the variability in dry bulk density observed during Proctor and kneading compaction processes could potentially exert a significant influence on the tensile strength of soils treated with lime [10]. In line with the study conducted by [7], reference [11] stated that despite being commonly used for quality control in field compaction, both the standard and modified Proctor tests do not accurately reflect the compaction effort exerted by modern field compactio nmachinery. Moreover, it is important to note that these methodologies cannot ascertain the compaction characteristics of soil materials beyond the parameters of MDD and OMC [11].

The study conducted by [12] investigated the hydromechanical characteristics of densely packed clay material used in dam core construction. Comparing drying-wetting paths between samples compacted at the standard Proctor optimum and those compacted according to the Proctor curve shows similarities in the wet side of the optimum [12]. Additionally, [13] investigated the compaction and strength properties of copper slag in relation to the influence of rice husk ash, lime, and cement through the implementation of Modified Proctor Compaction (MPC) and unconfined compressive strength (UCS) tests. The compaction density and water stability of Proctor specimens are better than those of vibration specimens, as Proctor compaction effectively facilitates the fragmentation of coarse-grained soil particles [14]. Furthermore, the present state of intelligent compaction technology in relation to asphalt materials is discussed in [15]. There is a discernible correlation between the maximum dry density and optimum moisture content, which can be observed by considering various compaction tests conducted at different compaction levels, in addition to the standard Proctor test [16].

The study conducted by [17] examined the compaction and degradation properties of CWRC (coal wash-rubber crumbs) mixtures intended for use as construction fill. The investigation consisted of five energy levels, spanning from standard to modified Proctor compaction [17]. It is important to note that the kaolin layer is situated on the drier end of the kaolin optimal moisture content, which is typically around 25 % as determined by the standard Proctor test. This specific moisture content is selected to ensure compatibility with the chilled mirror apparatus (WP4) and falls in the measurement range of over 100 kPa [18]. The use of a Proctor hammer for compaction, following the FLL and ASTM methods, resulted in an increased bulk density (at field capacity and dry conditions) and reduced water permeability of the substrate compared to the values obtained using the Australian Standard free-fall method of compaction [19]. The study carried out by [20] examines the relationship between moisture content and plasticity, specifically focusing on the impact of moisture on compaction. This observation is in line with the principles of the Proctor compaction theory, as the moisture content used was below the ideal range. It is important to note that higher moisture levels facilitate compaction, particularly the reduction in height. However, it is worth noting that all the moisture contents used for compaction were lower than OMC obtained from the modified Proctor test [20].

The Proctor compaction curves and G_max_ values of saline specimens studied by [21] showing varying levels of soil salinity showed a notable degree of similarity. According to a study conducted by [22], it was observed that implementing low compaction energy (600 KN-m/m^3^) after incorporating 8 % alum sludge, using a standard Proctor hammer, led to an improvement in California Bearing Ratio (CBR) values. Specifically, CBR values increased from 2.21, 3.44, and 5.51 to 6.33, 8.44, and 12.70 at 10, 30, and 65 blows respectively. The results indicated a notable improvement in soil strength, even when the compaction energy applied is relatively low [22]. Another study performed by [23] examines the shear strength and permeability characteristics of three lateritic soil samples obtained from local sources. The samples were treated exclusively through compaction, using energies ranging from 596 to 3576 kJ/m^3^. For comparison, the standard and modified Proctor energies were 596 and 2682 kJ/m^3^, respectively [23].

For Construction and demolition (C&D) waste application, the results of previous studies, as stated by [24], suggest that an increase in moisture content is associated with a decrease in interface shear strength, while a higher degree of compaction leads to increased interface shear strength [24]. In various Proctor tests, specifically ASTM D698–12 and ASTM D1557–12, the methodology consistently showed its efficacy in achieving significant reductions in void ratio at lower energy levels. In all instances, the Proctor method consistently yielded a maximum density that was lower than the maximum index density attainable through the standard vibratory method outlined in ASTM standards D4254–14 [25]. A study conducted by [26] investigated the compaction behavior of open-graded aggregates under simultaneous vibratory and impact compaction, also known as top-to-bottom compaction. In the study performed by [27], it was noted that Ir=100 % signifies the compaction of soil to its maximum dry density, as determined by a modified Proctor test. Moreover, when Ir exceeds 100 %, it signifies that soil has been compacted to a greater extent than what can be achieved through a modified Proctor test, showing a higher level of compaction effort [27]. Subsequently, an improvement using WGP (waste glass powder) was implemented [28]. Soil sample was mixed with a fixed proportion of 5 % cement and varying percentages of 2.5, 5, 7.5, and 10 % WGP. These modified soil samples were subjected to testing for Atterberg limits, Proctor's compaction, and CBR. These tests were conducted by [28] on both untreated and treated soil samples and based on their results of the conducted tests, the introduction of a combination of cement and WGP into clayey soil resulted in a decrease in both its liquid limit and plasticity index [28]. A study conducted by [29] provides a concise historical overview of the evolution of soil compaction, with a particular focus on the field of transport geotechnics. The examination of Proctor's curves and CBR was conducted by [29], and the corresponding current standards for field specifications were deliberated upon. The use of intelligent compaction is experiencing a growing trend due to the potential to improve compaction uniformity, which is a significant factor contributing to premature pavement failure [29].

B. Degree of compactness (DC)

The results by [30] examined the possibility of establishing a universal function that accurately characterizes the relationship between variable S and soil compactness across different soil textures. In this study, soil compactness was quantified using the degree of compactness, which represents the relative density of soil as the ratio of bulk density to a reference density. The study conducted by [30] reported that proctor density is a more effective measure of reference density compared to Håkansson reference density. The latter measure introduced a certain level of texture dependency in the relationship between S and degree of compactness. The results suggest that the reciprocal of S is a reliable indicator of soil compactness, thereby supporting the efficacy of S as a soil physical quality index [30].

In the study conducted by [31], the focus was on assessing soil physical quality, the hard-setting phenomenon (HDexter), and soil compactness in a set of 15 soils with varying gypsum content, which ranged from 30 to 301 g per kilogram (g kg^−1). Furthermore, the analysis of [32] focused on the impact of compaction effort, levels, and the degree of saturation. The results show that the compaction efforts implemented on the site are excessive, resulting in significant particle breakage. The explanation for the low levels of compaction achieved on certain sites, despite the high compaction efforts, can be understood when considering the degree of saturation [32]. Another study by [33] established a correlation between the compaction characteristics of natural soils at Proctor's energy level and the plastic limit. Subsequently, the plastic limit is adjusted to consider the percentage of fines that were smaller than 425 μm in the natural soils [33]. The compaction curves were determined by [34] through a series of modified Proctor compaction tests, as previously mentioned, and the outcomes acquired at various coarse aggregate contents (Cg) are graphically represented in [34]. The analysis showed that curves representing Cg values ranging from 0 % to 70 % show a singular peak pattern, closely resembling undiluted clayey soil. Therefore, the introduction of coarse gravels ranging from 0 % to 70 % into clayey soil does not appear to alter the configuration of compaction curves [34]. There exists a phenomenon explored by [35] wherein the rate of change in moisture content is minimal within the range of compaction degrees from 90 % to 96 %. However, a notable disparity in moisture content is observed as the compaction degree increases from 96 % to 99 % [35].

C. Optimum moisture content (OMC)

The results by [36] aimed to develop a vibratory soil compactor capable of generating outcomes that closely resemble those obtained through the Proctor test. The study found that Piarco sandy loam soil was the optimal soil type for the compactor, and the density values obtained closely matched those from the Proctor test [36]. The target density typically ranges from 97 % to 100 % of the dry density achieved in the laboratory through the modified proctor test (MPT), and the specific value in this range is determined by the applicable specification. In order to determine the optimal fluid content (OFC) of the investigated CIR mixtures, the RAP was subjected to modified Proctor tests in accordance with the EN 103–501 standard. The Proctor test results were adjusted to indicate a maximum dry density of 1944.00 kg/m^3^ for OMC of 5.75 % [37]. The categorization of soil based on Atterberg limits and sand coefficient uniformity has been found to improve the accuracy of compaction prediction in relation to organic carbon (OC) and sand plus clay (S + C) contents [38]. The Carazas and dropped ball tests, as described in [39], have shown that the optimal range for moisture content in manufacturing processes typically falls between 12 % and 16 %. This range is considered ideal for ensuring appropriate adaptability during the shaping phase of manufacturing processes. This specific range is crucial for achieving appropriate adaptability during the shaping phase through compaction [39].

As noted in the study by [40], concerning OMC, it was observed that different moisture content on either side of OMC can result in the same density, but do not exhibit the same modulus. This suggests that while density may remain consistent, the mechanical properties and behavior of the material can vary depending on whether the moisture content is above or below OMC. The findings comprehensively investigated the impact of moisture content on the values of intelligent compaction measurement values (ICMV) and *in-situ* point measurements through the use of both laboratory tests and *in-situ* experiments [40]. The investigation by [41] gum Arabic impact on MDD and OMC of laterite soil was assessed for potential use as a binding agent for soil block stabilization. The results showed that the maximum dry density exhibited a decrease from 1883 kg/m^3^ to 1693 kg/m^3^ following the incorporation of gum Arabic in laterite soil at concentrations ranging from 0 % to 10 %. The addition of gum Arabic in the range of 0 % to 10 % resulted in an increase in OMC from 14.88 % to 18.38 % [41]. OMC of sandy clay soil is lower when reinforced with basalt fiber compared to sandy clay soil without reinforcement. The incorporation of an optimal percentage of Basalt fiber into sandy clay soil has the potential to reduce the optimum moisture conte OMC nt in comparison to soil that does not contain Basalt fiber [42]. In the study by [43], soil sample is subjected to compaction within a conventional metallic mold. Subsequently, a cylindrical plunger is introduced into soil at a predetermined rate of penetration. A remold specimen is prepared using a specific MDD and OMC in order to conduct standard or modified Proctor compaction tests [43].

In the study by [43], a soil sample undergoes compaction within a conventional metallic mold. Subsequently, a cylindrical plunger is introduced into the soil at a predetermined rate of penetration. A remold specimen is prepared using specific MDD and OMC values to conduct standard or modified Proctor compaction tests [43].

According to the study conducted by [44], the proposed permanent deformation (PD) has shown its ability to accurately predict the impact of compaction moisture content on the long-term resistance to PD of fine soils. The suggested procedure shows potential as a valuable tool for material selection [44]. However, when the stabilizer level was increased from 10 % to 20 %, the results suggested that the formulae with higher moisture content showed superior strength [45]. According to the regression analysis conducted by [46] on the data obtained from the Light Weight Deflectometer modulus results, it was found that all three linear terms (compaction level, moisture content, and drop mass), as well as the interaction term between compaction and moisture, were statistically significant at a confidence level of 90 % [46]. Regardless of the observed impact of soil moisture on structure formation in various soil types, in the range of compaction and moisture levels tested, it can be inferred that compaction has a significantly greater influence on effective permeability compared to soil moisture. This influence is primarily attributed to the redistribution of pore sizes, specifically the reduction of wider-diameter pores [47]. This is in line with the study by [48] that assessed the influence of sample size on the mechanical properties of cement-stabilized sandy soil, specifically focusing on the porosity/cement ratio (η/Civ), while considering optimal compaction conditions. In the study conducted by [49], experimental investigations were carried out to assess how varying moisture contents affect the properties of hydrated lime fly ash (HLF) bricks when different mixing sequences were used. The objective of the research was to determine the most efficient method for producing HLF bricks under specific parameters. The objective was to determine the most efficient method for producing HLF bricks under the specified parameters. The bricks, which had a moisture content of 15 %, were manufactured using a two-stage mixing process. It was observed that these bricks exhibited improved mechanical strength and durability characteristics [49]. The criterion for moisture content in vibration compaction is widely accepted to be the OMC of High-Speed Railway Graded Aggregate (HRGA) materials, which is determined using the Heavy Hammer Compaction Method (HHCM). The results suggest that the Critical Moisture Content (CMC) index can be used as a standardized measure for determining OMC in Low Volume Cementitious Materials (LVCM). Additionally, it is observed that HRGA materials with CMC show favorable strength and load-bearing capabilities [50].

## Experimental setup

### Test procedures

This experiment includes a series of soil physical and mechanical properties tests carried out in soil mechanics laboratory. The purpose is to determine the physical and mechanical properties of the original soil both without and with the addition of a soil stabilizing agent. Subsequently, the laboratory testing procedures adhere to applicable standards. Soil samples used consist of both undisturbed soil and disturbed soil obtained from certain locations using the test pit method. It is recommended that undisturbed soil samples be wrapped in plastic or aluminum foil coded and then stored in a place that is not hot (humid). The measurement of soil moisture content is a crucial parameter in soil testing.

Laboratory tests of unstabilized soil include: moisture content test (w), soil unit weight test (γ), specific gravity (Gs), grain gradation analysis, atterberg limits, and compaction (modified proctor). Meanwhile, laboratory test of stabilized soil includes atterberg limits and compaction (modified proctor).

### Moisture content test (w)

**Fig. 1:**
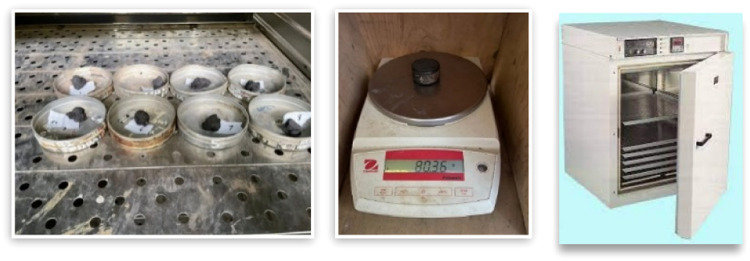
Moisture content testing.

Moisture content is the ratio between the weight of water contained in soil and the weight of dry soil particles expressed in percentage (%), using standard ASTM D 2216–80. Subsequently, moisture content is tested using undisturbed soil samples, and the value is obtained by calculating the weight of the wet soil minus the weight of the dry soil divided by the weight of the cup, which the process was described in Fig. 1.

The formula for soil moisture content is as follows:

(1) $$\text{Moisture}\;\text{content}(w)=\frac{Ww}{Ws}=\frac{\left(W1-W2\right)}{W2-W3}\cdot 100\%$$

Where:

*w*= Moisture content (%)
W_1_= Weight of saucer + wet soil (gr)
W_2_= Saucer weight + dry soil (gr)
W_3_= Weight of cup (gr)
W_w_= water weight
W_s_= dry soil weight

### Soil unit weight test (γ)

**Fig. 2:**
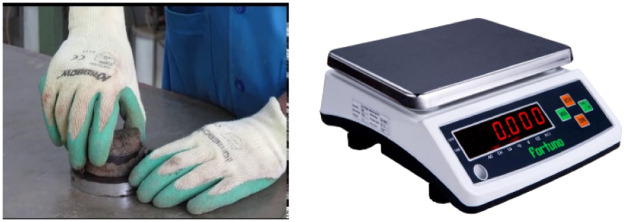
Soil unit weight testing.

The unit weight of wet soil is the ratio between the weight of the original soil and the volume of the original soil, expressed in gr/cm^3^, using standard ASTM D 2937–83. This test uses undisturbed soil samples with ring-shaped specimens. Subsequently, the value of soil unit weight (γ) is obtained by calculating the weight of the wet soil (W) divided by the volume of the ring (V), which the process was described in Fig. 2.

In order to create soil ring, it is necessary to perform the following procedures: cleansing the ring, determining the volume of the ring (V), and measuring its weight (W_1_). Additionally, it is important to exercise caution when positioning the pointed section of the ring onto the surface of the ground, ensuring that soil can seamlessly conform to the contours of the ring. It is also essential to meticulously sever and level both facets of the ring using a knife, to avoid any perforations. In the event of a diminutive aperture, it is imperative to mend it using identical soil. Ultimately, it is imperative to thoroughly cleanse the residual soil adhered to the external surface of the ring, followed by the subsequent measurement of the weight of soil-contained ring (W_2_). The calculation of soil content weight can be calculated as follows:

(2) $$\text{Weight}\;\text{of}\;\text{soil}\;\text{content}\;\left(\gamma_{\text{wet}}\right)=\frac{\left(W2-W1\right)}{V}\left[\text{gr}/cm^{3}\right]$$

where:

W_1_ = ring weight (gr)
W_2_ = ring weight + soil (gr)
*V* = Ring volume (cm^3^)

### Specific gravity (Gs)

**Fig. 3:**
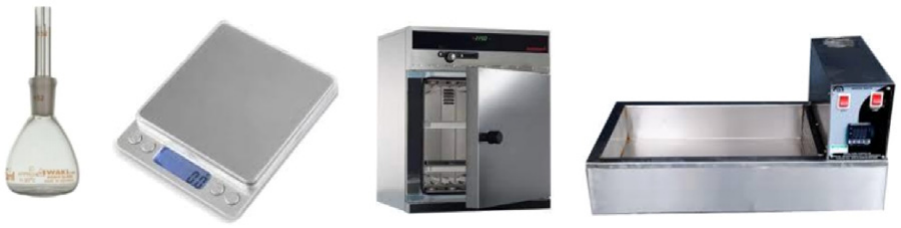
Gs test equipment.

Gs of soil is a fundamental property representing the mass of dry soil that occupies a given space in soil layer. It is expressed as the mass per unit of dry soil, using standard ASTM D 854–33. The weight of W_4_ is determined in Fig. 3 by using a table containing weight values of W_4_ and shows the correlation between Gs of water and the correction factor denoted as k. In order to assess the uniformity of material and soil properties, a pycnometer is used for measuring soil density, particularly targeting samples that can pass through a filter with a size of either 4.75 mm (no. 4) or 2.00 mm (no. 10). In the context of Gs testing, two distinct categories of samples are used, namely dry test samples and wet test samples. The dry test sample is subjected to a drying process for at least 12 h, or until its weight remains constant when exposed to an oven set at a temperature of 110 ± 5 °C (230 ± 9°F). Subsequently, the sample is then transferred into a pycnometer filled with distilled water. Before being placed in the measuring bottle, it is advisable to immerse the wet soil sample in distilled water for 24 h. This process allows soil to become thoroughly saturated and dissolved in the water, ensuring accurate measurements when determining Gs. Additionally, a pycnometer or measuring bottle containing a measured sample is immersed in a bath and filled with distilled water. The correction factor (k) is subsequently computed, enabling the determination of Gs of soil through the utilization of a prescribed formula and detailed in Table 1. The approach is a comprehensive methodology that consists of succinct and precise steps and instructions, thereby facilitating the laboratory testing of soil Gs. According to the Indonesian National Standard, soil density test method mandates that the density test is performed using a double system, often referred to as “Duplo.” In this approach, two separate tests are conducted, and the results obtained from both tests are subsequently averaged. This practice helps improve the reliability and accuracy of the density measurements for soil. The significance of this testing methodology lies in its ability to ascertain the composition and properties of soil, as Gs of soil plays a crucial role in influencing various key factors including carrying capacity, water retention, and soil permeability. Therefore, the use of soil Gs testing can aid in assessing the appropriateness of a given location for construction or other related activities.

(3) $$W_{4}=W_{25}\cdot k$$

**Table 1:** Relationship between relative density of water and conversion factor *k* in temperature.

ºT (Temperature)	The relationship of the relative density of water	Correction factor *k*
18	0. 9,986,244	1. 0016
19	0. 9,984,347	1. 0014
20	0. 9,982,343	1. 0012
21	0. 9,980,233	1. 0010
22	0. 9,978,019	1. 0007
23	0. 9,975,192	1. 0005
24	0. 9,973,286	1. 0003
25	0. 9,970,770	1. 0000
26	0. 9,968,156	0. 9997
27	0. 9,965,451	0. 9995
28	0. 9,962,652	0. 9992
29	0. 9,939,761	0. 9989
30	0. 9,956,780	0. 9986

Where:

W4= Weight of pycnometer + water + close after correction (gr)
W25= Weight of pycnometer + water + cover at 25 °C (gr)
K= Correction factor for temperature

Calculate Gs of soil with the formula below:

(4) $$G_{S}=\frac{G_{L}\left(W2-W1\right)}{\left(W4-W1\right)-\left(W3-W2\right)}$$

Where:

Gs = Specific gravity of soil
G_L_ = specific gravity of the liquid used
W1 = Weight of pycnometer + cover
W2 = Weight of pycnometer + soil sample + cover
W3 = Weight of pycnometer + soil sample + water + lid

### Soil grain gradation test

**Fig. 4:**
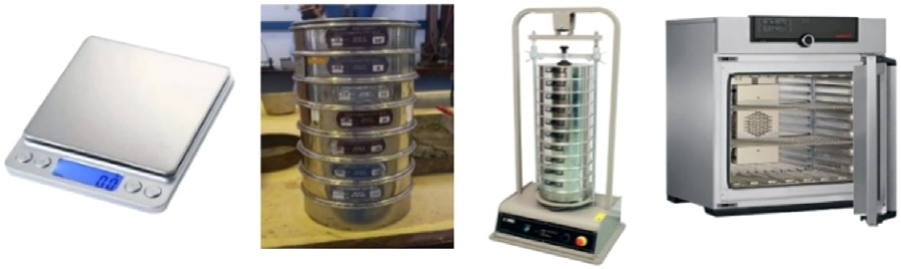
Soil grain gradation test equipment.
b. Hydrometer analysis

The particles that form soil structure have various sizes and shapes, both in cohesive and non-cohesive soil. Subsequently, soil properties are primarily influenced by the size and distribution of its grains. Therefore, in soil mechanics, grain size analysis is frequently used as a basis for soil classification. Grain gradation is the grouping of coarse and fine grains into a combined composition which is reviewed based on the sieve and hydrometer, the results of the analysis are carried out to determine the gradation limits of soil grains, using standard ASTM D 422–72. This Granule Analysis Test is carried out in two ways:

a. Sieve analysis: for coarse-grained soil content (sand, gravel) such depicted in Fig. 4.

**Fig. 5:**
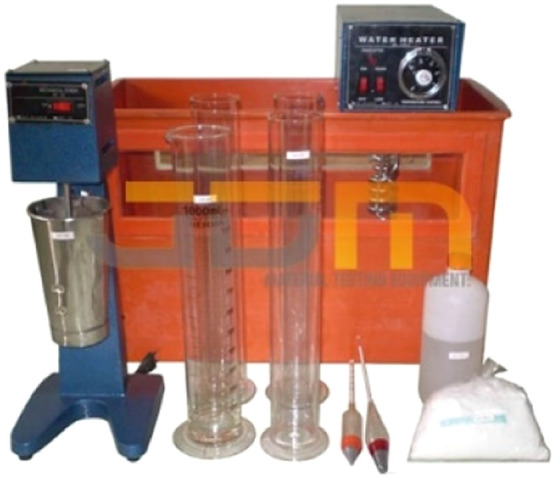
Hydrometer analysis test equipment.

To determine the grain size distribution of soil passing through sieve no. 200. Hydrometer analysis is carried out based on the principle of sedimentation of soil grains in water using equipment in Fig. 5. When a soil sample is dissolved in water, soil particles will settle at different rates depending on the shape, size, and weight.

The formula used :

(5) $$R_{C}=R_{a}-Z_{C}+C_{t}$$

where:

R_C_ = Corrected hydrometer reading
R_a_ = hydrometer reading at test
Z_c_ = Correction to zero hydrometer
C_t_ = Correction for temperature (Table 2)

**Table 2:** Correction (Ct) to temperature.

Temperature (°C)	Ct
15	−1.10
16	−0.90
17	−0.70
18	−0.50
19	−0.30
20	0.00
21	0.20
22	0.40
23	0.70
24	1.00
25	1.30
26	1.65
27	2.00
28	2.50
29	3.05
30	3.80

(6) $$\%\;\text{pass}=\frac{R_{c}.a}{W_{s}}.100\%$$

Where:

Rc= Corrected hydrometer reading
*a*= correction to Gs = 2.65 (Table 3)

**Table 3:** Correction (a) for specific gravity (Gs).

Specific Gravity (Gs)	Correction factor (a)
2.85	0.96
2.80	097
2.75	0.98
2.70	0.99
2.65	1.00
2.60	1.01
2.55	1.02
2.50	1.04
Ws= Weight of dry specimen

(7) $$R=R_{a}+1$$

Where:

*R*= Hydrometer reading corrected only by the meniscus
Ra= Hydrometer reading during testing
*L*= Distance traveled by the granules (Table 4)

**Table 4:** The value of L (effective depth).

Hydrometer reading corrected by meniscus (R)	Effective depth (L)	Hydrometer reading corrected by meniscus (R)	Effective depth (L)	Hydrometer reading corrected by meniscus (R)	Effective depth (L)
0	16.3	21	12.9	42	9.4
1	16.1	22	12.7	43	9.2
2	16.0	23	12.6	44	9.1
3	15.8	24	12.4	45	8.9
4	15.6	25	12.2	46	8.8
5	15.5	26	12.0	47	8.6
6	15.3	27	11.9	48	8.4
7	15.2	28	11.7	49	8.3
8	15.0	29	11.5	50	8.1
9	14.8	30	11.4	51	7.9
10	14.7	31	11.2	52	7.8
11	14.5	32	11.1	53	7.6
12	14.3	33	10.9	54	7.4
13	14.2	34	10.7	55	7.3
14	14.0	35	10.5	56	7.1
15	13.8	36	10.4	57	7.0
16	13.7	37	10.2	58	6.8
17	13.5	38	10.1	59	6.6
18	13.3	39	9.9	60	6.5
19	13.2	40	9.7		
20	13.0	41	9.6

(8) $$v=\frac{L}{t}$$

(9) $$D=\sqrt{\frac{L}{t}}$$

Where:

*v* = speed of grain settling
*t* = observation time
*D* = grain diameter
*K* = correction for temperature and Gs (Table 5)

**Table 5:** K values (correction for temperature and Gs).

Temp. °C	Specific Gravity (Gs)							
	2.50	2.55	2.60	2.65	2.70	2.75	2.80	2.85
16	0.0151	0.0148	0.0146	0.0144	0.0141	0.0139	0.0137	0.0136
17	0.0149	0.0146	0.0144	0.0142	0.0140	0.0138	0.0136	0.0134
18	0.0148	0.0144	0.0142	0.0140	0.0138	0.0136	0.0134	0.0132
19	0.0145	0.0143	0.0140	0.0138	0.1360	0.0134	0.0132	0.0131
20	0.0143	0.0141	0.0139	0.0137	0.0134	0.1330	0.0131	0.0129
21	0.0141	0.0139	0.0137	0.0135	0.0133	0.1310	0.0129	0.0127
22	0.0140	0.0137	0.0135	0.0133	0.0131	0.0129	0.0128	0.0126
23	0.0138	0.0136	0.0134	0.1320	0.0130	0.0128	0.0126	0.0124
24	0.0137	0.0134	0.0132	0.0130	0.0128	0.0126	0.0125	0.0123
25	0.0135	0.0133	0.0131	0.0129	0.0127	0.0125	0.0123	0.0122
26	0.0133	0.0131	0.0129	0.0127	0.0125	0.0124	0.0122	0.0120
27	0.0132	0.0130	0.0128	0.0126	0.0124	0.0122	0.0120	0.0119
28	0.0130	0.0128	0.0126	0.0124	0.0123	0.0121	0.0119	0.0117
29	0.0129	0.0127	0.0125	0.0123	0.0121	0.0120	0.0118	0.0116
30	0.0128	0.0126	0.0124	0.0122	0.0120	0.0118	0.0117	0.0115
*L* = distance traveled by the grain

### Atterberg limits

Fine-grained soil containing clay minerals is very sensitive to changes in moisture content. Atterberg established specific points in the form of liquid, plastic, and shrinkage limits. By knowing soil consistency value, the plasticity properties of soil can be known. The properties of plasticity are expressed by the price of the elasticity index (Plasticity Index) which is the difference between the value of the liquid limit moisture content and the value of the plastic limit moisture content (IP = LL-PL). A higher PI value shows greater sensitivity to moisture content changes, increased shrinkage, and a substantial impact on soil carrying capacity and strength. Subsequently, soil samples with grains larger than sieve No. 40 (0.425 mm) should be dried and filtered. Take 200 g of specimens that pass sieve No.40 (0.425 mm).

**Fig. 6:**
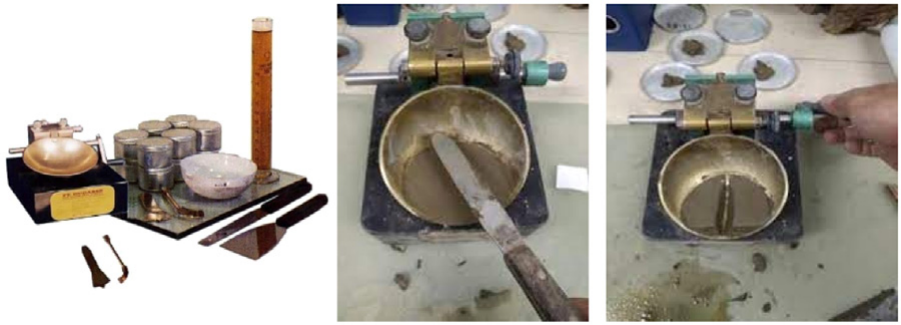
Atterberg limits test equipment.

To determine the value of the consistency limits of fine-grained soils by considering soil moisture content. These limits are the liquid limit (LL) and plastic limit (PL), using standard ASTM D 4318 – 84 which use equipment in Fig. 6.

### Proctor modified test

To process a damp soil sample from the field, follow these steps, dry it in an aerated way (air dry) or an oven with a maximum temperature of 60 °C. Then separate the lumps of soil by pounding them with a rubber mallet. The crushed soil is sieved through a No.4 sieve (#4.75 mm). The results of each sieve were weighed as much as 5 kg, each of 6 pieces. Gradually mix soil weight results with water, stirring until uniform, and then store the mixture in a labeled bucket for 24 h.

Soil compaction modified test is a laboratory method for determining OMC value of a certain soil type and MDD value using standard ASTM D-1556 and the result can be seen in Table 6.

**Table 6:** Types of compactions in the laboratory.

Test	Modified proctor			
Method	A	B	C	D
Diameter [mm]	102	152	102	152
Mold height [mm]	116	116	116	116
Mold volume [cm^3^]	943	2124	943	2124
Pounder weight [kg]	4.54	4.54	4.54	4.54
Fall height [cm]	30.5	30.5	30.5	30.5
Number of layers	5	5	5	5
Number of collisions per layer	25	56	25	56
The material passes through the filter [mm]	4.75	4.75	4.75	4.75

### Monitoring analysis of original soil without sand and lime

Step 1: Moisture content test (w)

Moisture content test is performed on three undisturbed soil samples. Moisture content values (w) are shown in Table 7 where the average value of the 3 specimens is 53.70 %.

**Table 7:** Determination of moisture content (ASTM D2216–80) at the point depth 1 m.

Cup Number	symbol	unit	1	2	3
Cup weight	(W_3_)	(gr)	9.56	9.78	9.99
Saucer weight + wet-soil	(W_1_)	(gr)	13.65	13.75	13.82
Saucer weight + dry-Soil	(W_2_)	(gr)	12.27	12.35	12.45
Moisture content	[W_w_=W_1_-W_2_]	(gr)	1.38	1.4	1.37
Weight of dry soil	[Ws=W_2_-W_3_]	(gr)	2.71	2.57	2.46
Moisture content (w)	[*w*=W_w_ + W_s_ . 100 %]	(%)	50.92	54.47	55.69
Average moisture content	(W_avg_)	(%)		53.7	

*Step 2: Soil unit weight test (γ)*

Soil content weight is determined through the testing of three specimens of undisturbed soil (UDS), as presented in Table 8 which shows the average weight value of wet soil content (γ) of the 3 specimens is 1.69 gr/cm^3^.

**Table 8:** Soil weight test (γ) using ASTM D2937–83 at the point depth 1 m.

Mold No.	symbol		unit	1	2	3
Mold weight + wet Soil	(W_2_)		(gr)	73.56	76.92	85.91
Mold weight	(W_1_)		(gr)	31,95	36,65	34,57
Wet soil weight	(W_2_)		(gr)	41.61	40.27	51.34
Mold diameter	[D]		(cm)	4.04	4.01	4.02
Mold height	[t]		(cm)	1.9	2.1	2.2
Mold volume	[*V*=¼.π.D².t]		(cm³)	24.36	26.52	27.92
Weight of soil contents	[*ϒ*ₜ=*W*ₜ÷V]		(gr/cm³)	1.71	1.52	1.84
Average wet soil fill weight		(gr/cm³)		1.69		

Step 3: Specific gravity (Gs)

Moisture content test is performed on three undisturbed soil samples. Moisture content values (w) are shown in Table 9 for soil Gs which is 2.69.

**Table 9:** Specific gravity (Gs) using ASTM D854–83 at the point depth 1 m.

Cup Number	symbol	unit	1	2	3
Weight of pycnometer + cap	(W_1_)	(gr)	28.69	27.04	27.31
Weight of pycnometer + dry soil + cap	(W_2_)	(gr)	38.69	37.04	37.31
dry soil weight	(W_t_=W_2_-W_1_)	(gr)	10	10	10
*pycnometer weight + dry soil + water + t*	(W_3_)	(gr)	84.48	84.15	84.84
pycnometer weight + water + cap	(W_4_)	(gr)	78.32	78.05	78.66
Temperature		( °C)	27	27	27
Temperature correction factor	(K)		0.9995	0.9995	0.9995
Corrected weight of pycnometer + water	(W_5_)		78.28	78.01	78.62
Soil specific gravity	(W_2_-W_1_) [(W_5_-W_1_)-(W_3_-W_2_)]		2.70	2.73	2.65
Average soil specific gravity			2.69		

As shown in Table 9, the average Gs value for the three specimens is 2.62.

Step 4: Grain gradation analysis

**Fig. 7:**
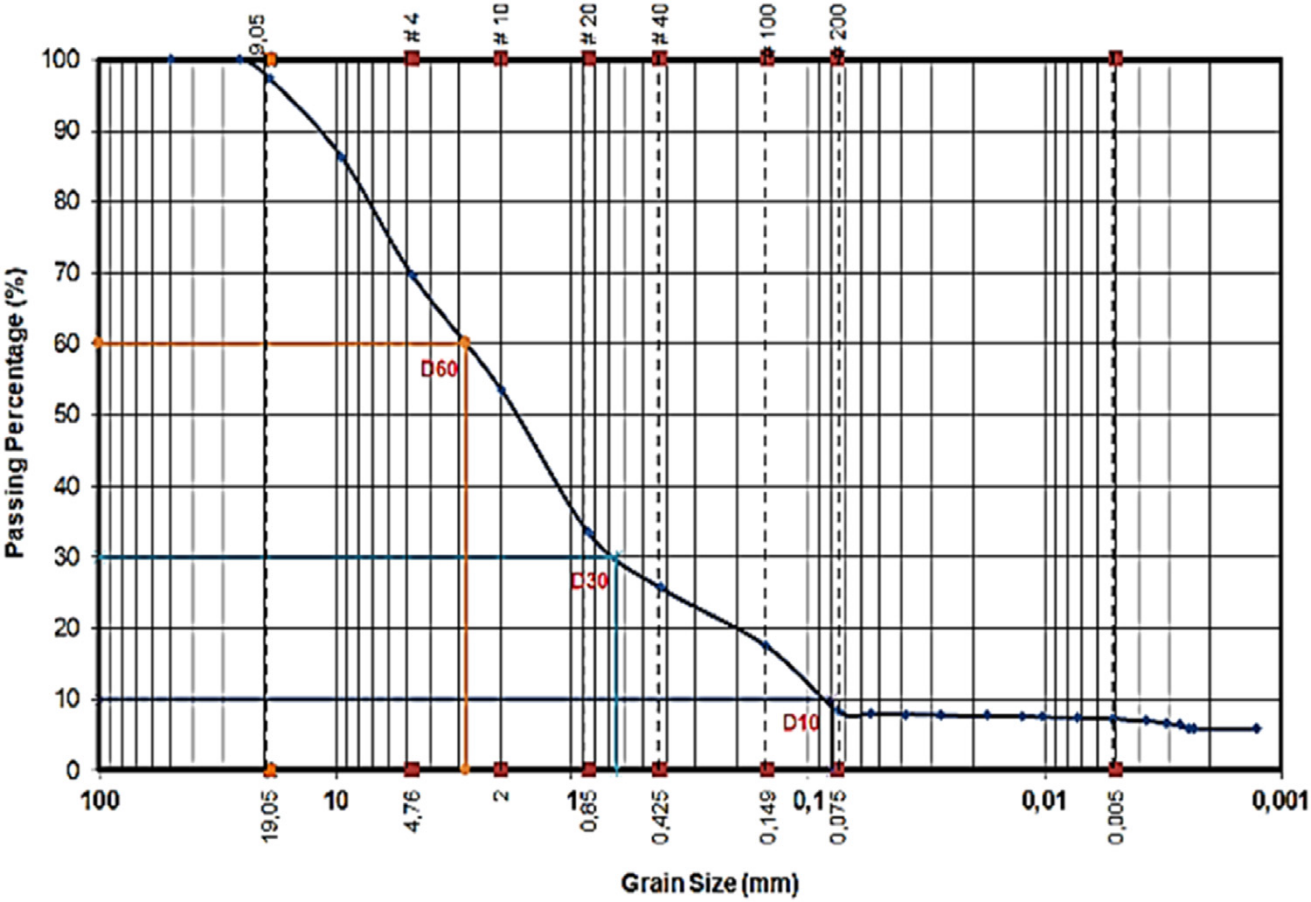
Grain size distribution curve.

Fig. 7 shows the grain size distribution graph, which is obtained through the analysis of sieving and hydrometer techniques. The data show that D10 has a value of 0.08, D30 has a value of 0.65, and D60 has a value of 2.80. Based on the measurements, the uniformity coefficient (Cu) can be computed as 35, while the gradation coefficient (Cc) is determined to be 1.886. The AASHTO classification system yields classification group A-2–7, which corresponds to a soil composition consisting of silty gravel or clay and sand.

Step 5: Atterberg limits

**Fig. 8:**
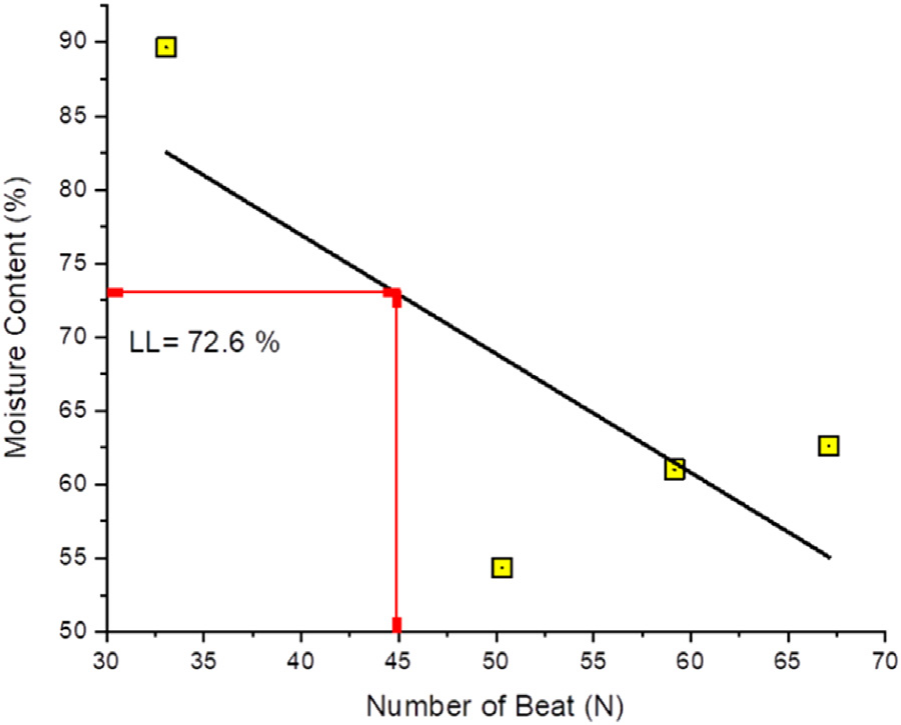
Liquid limit (LL) determination.

The assessment of Atterberg limits includes the use of four soil specimens that are capable of passing through a filter with a mesh size of #40 (#40). The test quantifies moisture content (w) and the quantity of impacts (N) for each sample. Subsequently, the provided data to construct Table 10 and linear graph in Fig. 8 to determine the liquid limit (LL) corresponding to a knock value (N) of 25. Fig. 8 shows a graphical representation showing the process of determining the liquid limit value (LL) which has a natural LL of 72.6 %.

**Table 10:** Liquid Limit (LL) original soil test results using ASTM D4318–84.

Cup No.	A.1	A.2	B.1	B.2	C.1	C.2	D.1	D.2
Weight of cup + wet soil [W_1_] (gr)	12.33	12.23	12.56	12.91	12.76	12.63	12.42	13.62
Weight of cup + dry soil [W_2_] (gr)	11.55	11.23	11.85	11.19	11.56	11.33	11.27	11.64
Weight of cup [W_3_] (gr)	9.89	9.96	9.55	9.31	9.03	9.23	9.61	9.85
Water weight [W_w_ = W_1_-W_2_] (gr)	0.78	1.00	0.71	1.72	1.20	1.30	1.15	1.98
Dry soil weight [W_s_ = W_2_-W_3_] (gr)	1.66	1.27	2.30	1.88	2.53	2.10	1.66	1.79
Moisture content [*w* = Ww/Ws x 100 %] (%)	46.99	78.74	30.87	91.49	47.43	61.90	69.28	110.61
Average Moisture content (W_avg_) (%)	62.86		61.18		54.67		89.95	
Number of blows (N)	43		35		28		18

Table 11 shows that the average plastic limit (PL) of the two samples is 51.66 %. Plasticity index value (IP) = LL - PL = 72.6 % - 51.66 % = 20.94 %.

**Table 11:** Determination of plastic limit (PL).

Cup Number	symbol	unit	1	2
Saucer weight + wet soil	(W_1_)	(gr)	13.44	14.15
Saucer weight + dry soil	(W_2_)	(gr)	12.69	12.89
Cup weight	(W_3_)	(gr)	10.37	11.12
Water weight	[W_w_=W_1_-W_2_]	(gr)	0.75	1.25
Dry soil weight	[Ws=W_2_-W_3_]	(gr)	2.32	1.77
Moisture content	[*w*=W_w_/ W_s_ . 100 %]	(%)	32.54	70.76
	(W_avg_)	(%)	51.66	

Step 6: Compaction (modified proctor)

The outcomes of modified compaction tests conducted on natural soils, both with and without sand and lime stabilization, are presented in Table 12 which shows the MDD and OMC values. A modified compaction test was conducted on disturbed soil samples (DS) that passed through a #4 sieve. This test included six specimens, each subjected to curing periods of 1 day, 7 days, and 14 days.

**Table 12:** MDD and OMC values original soil results.

Compaction testing	Curing time	Original Soil (OS)
MDD (gr/cm^3^)	1	1.52
	7	1.64
	14	1.70
OMC (%)	1	22.55
	7	19.60
	14	21.78

### Monitoring analysis original soil with sand and lime

Step 1: Atterberg limits

Four test specimens are used to determine the Atterberg limits of soil passing through a #40 sieve, which had been stabilized with a mixture of sand and lime. In the tests, the following were determined, moisture content (w) and the number of blows (N) on each test object, then a graph was drawn to determine the liquid limit value (LL). Table 13 shows the results of the original soil Atterberg limits test with sand + lime stabilization.

**Table 13:** Results of the stabilization Atterberg limits test.

Composition of a mixture of Sand and Lime	Atterberg Limits		
	LL	PL	IP
Original soil (OS)	72.60	51.66	20.94
OS + 20 % Sand + 5 % Lime	62.71	49.59	13.12
OS + 20 % Sand + 10 % Lime	56.88	47.04	9.84
OS + 20 % Sand + 15 % Lime	60.48	55.28	6.89
OS + 20 % Sand + 20 % Lime	58.50	49.69	8.82

**Fig. 9:**
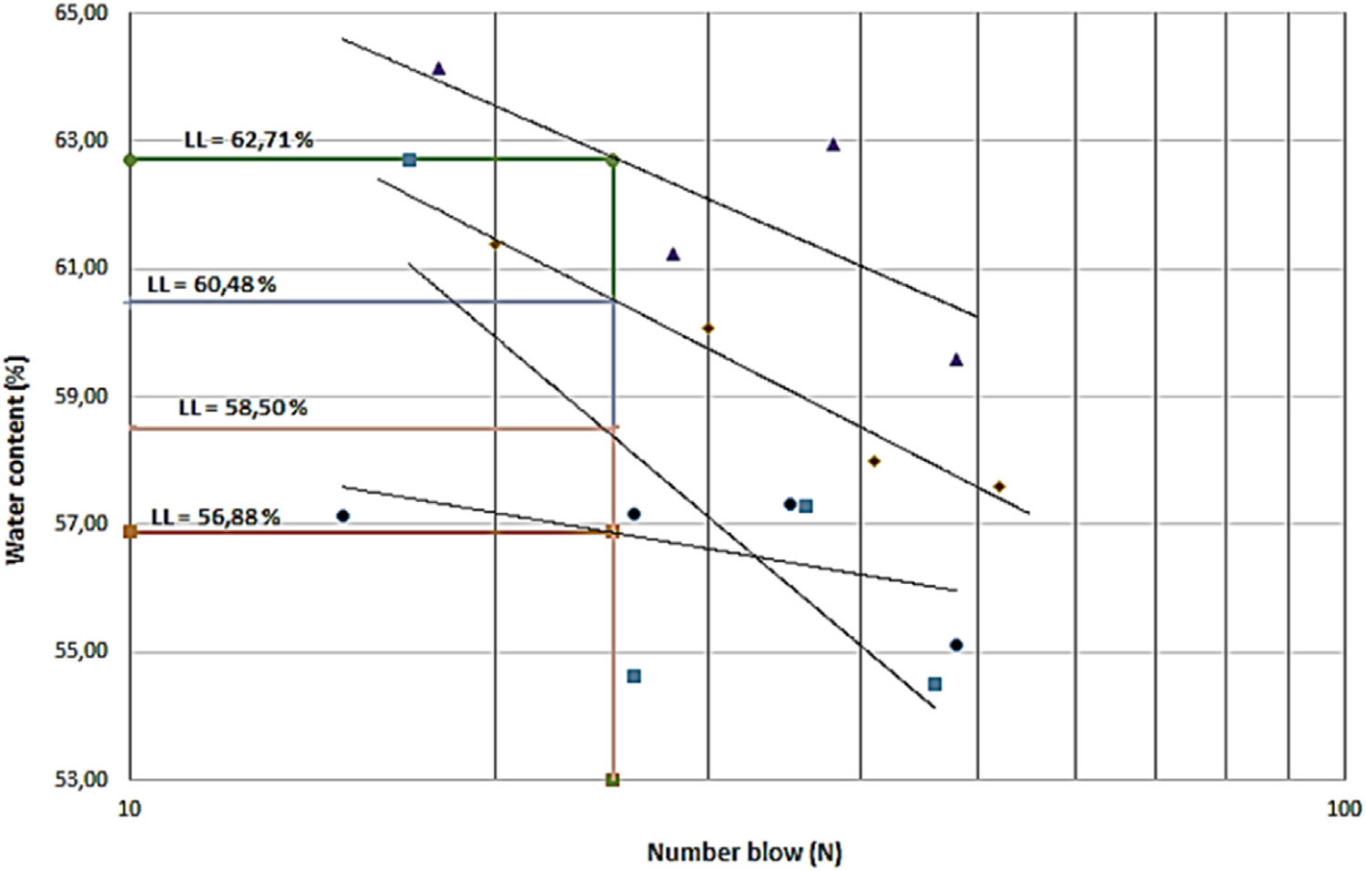
Liquid limit (LL) curve with stabilization variations.

Table 13 shows the value of the liquid limit (LL), plastic limit (PL), and plasticity index (IP) value for each composition of the variation of sand + lime. The results of the analysis is found in Fig. 9 with the addition of sand + lime to the original soil tend to show a decrease in the value of the plasticity index (IP) from the plasticity index (IP) value of the original soil without stabilization.

Step 2: Compaction (Modified proctor)

A compaction test was conducted using modified procedures with disturbed soil samples (DS) that passed through a #4 sieve. Approximately 6 specimens were stabilized by a mixture of sand and lime. Table 14 shows MDD and OMC values.

**Table 14:** Modified compaction test with stabilization results.

Mixed composition	Curing time (day)	MDD (gr/cm^3^)	OMC (%)
Original soil (OS)	1	1.522	22.80
	7	1.641	19.60
	14	1.697	21.78
OS + 20% Sand + 5% Lime	1	1.719	19.43
	7	1.703	20.47
	14	1.775	19.16
OS + 20% Sand + 10% Lime	1	1.665	20.25
	7	1.867	24.25
	14	1.785	21.45
OS + 20% Sand + 15% Lime	1	1.657	25.85
	7	1.739	20.16
	14	1.869	20.61
OS + 20% Sand + 20% Lime	1	1.748	21.98
	7	1.573	22.45
	14	1.705	22.27

**Fig. 10:**
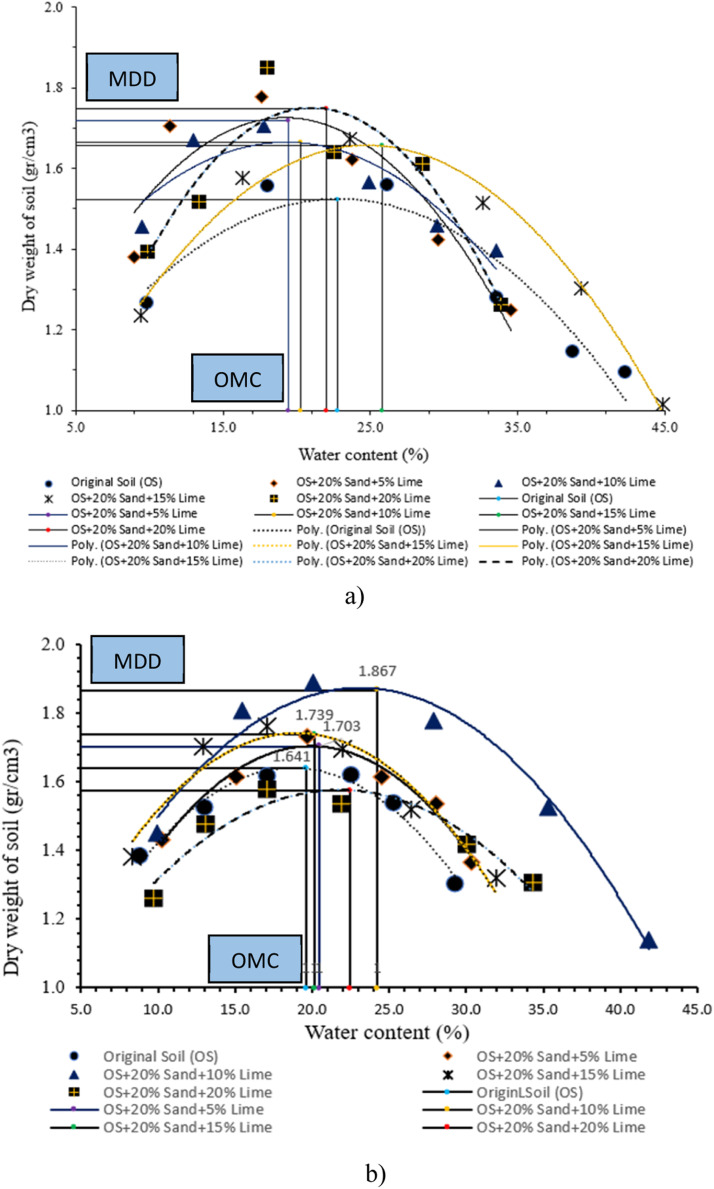

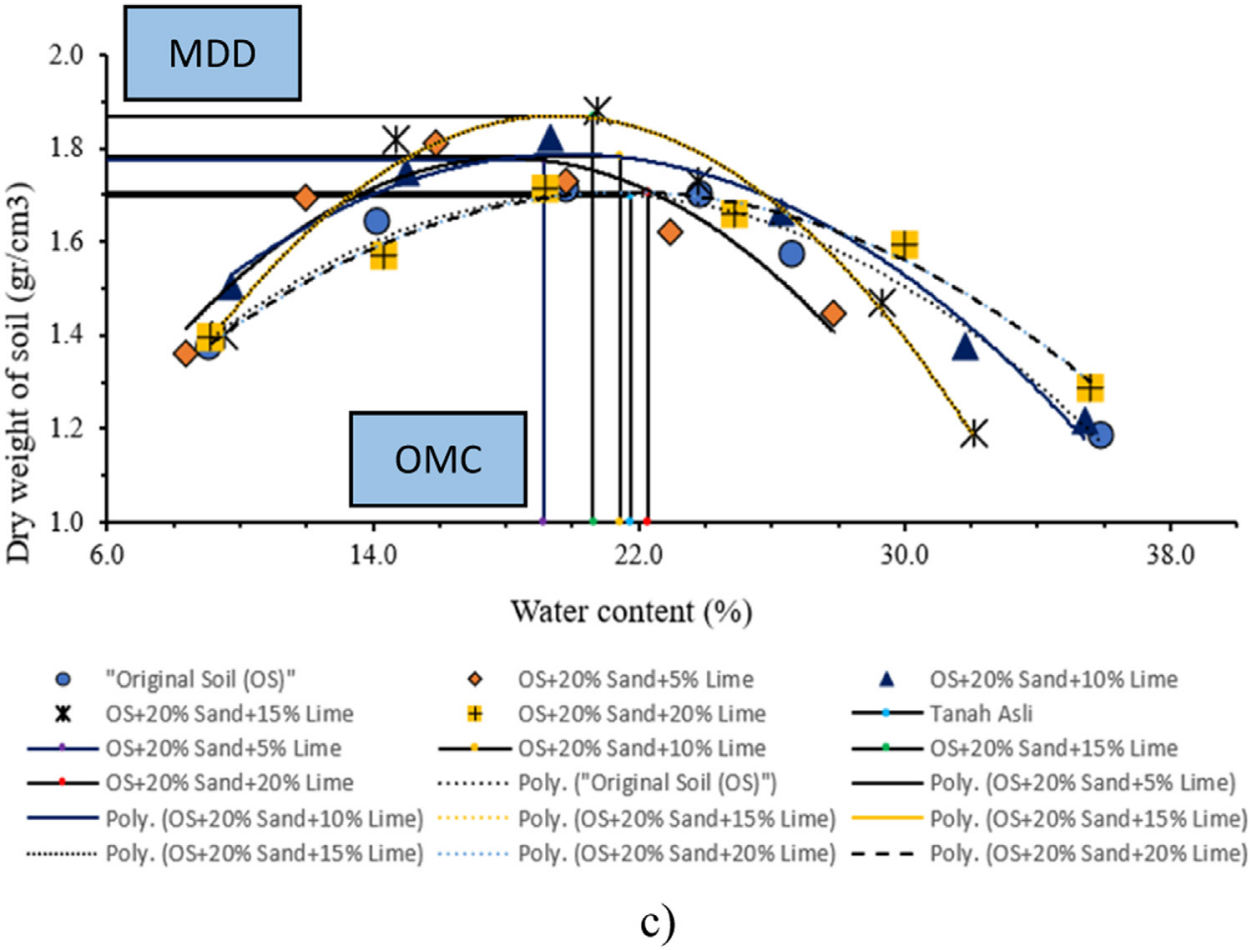
Modified compaction at (a) 1-day (b) 7-day (c) 14-day curing.

The observed trend in Table 14 shows the results of the modified compaction test at each curing time of the original soil without and with sand and lime stabilization, showing that MDD shows an upward trajectory, while OMC shows a downward trend in comparison to the initial unstable soil conditions. Additionally, MDD observed during the initial day of MDD test conducted on unstabilized natural soil is recorded as 1.522 gr/cc. However, this value increases to 1.719 gr/cc upon stabilization with a mixture comprising 20 % sand and 5 % lime. Please refer to Fig. 10a–c.

**Fig. 11:**
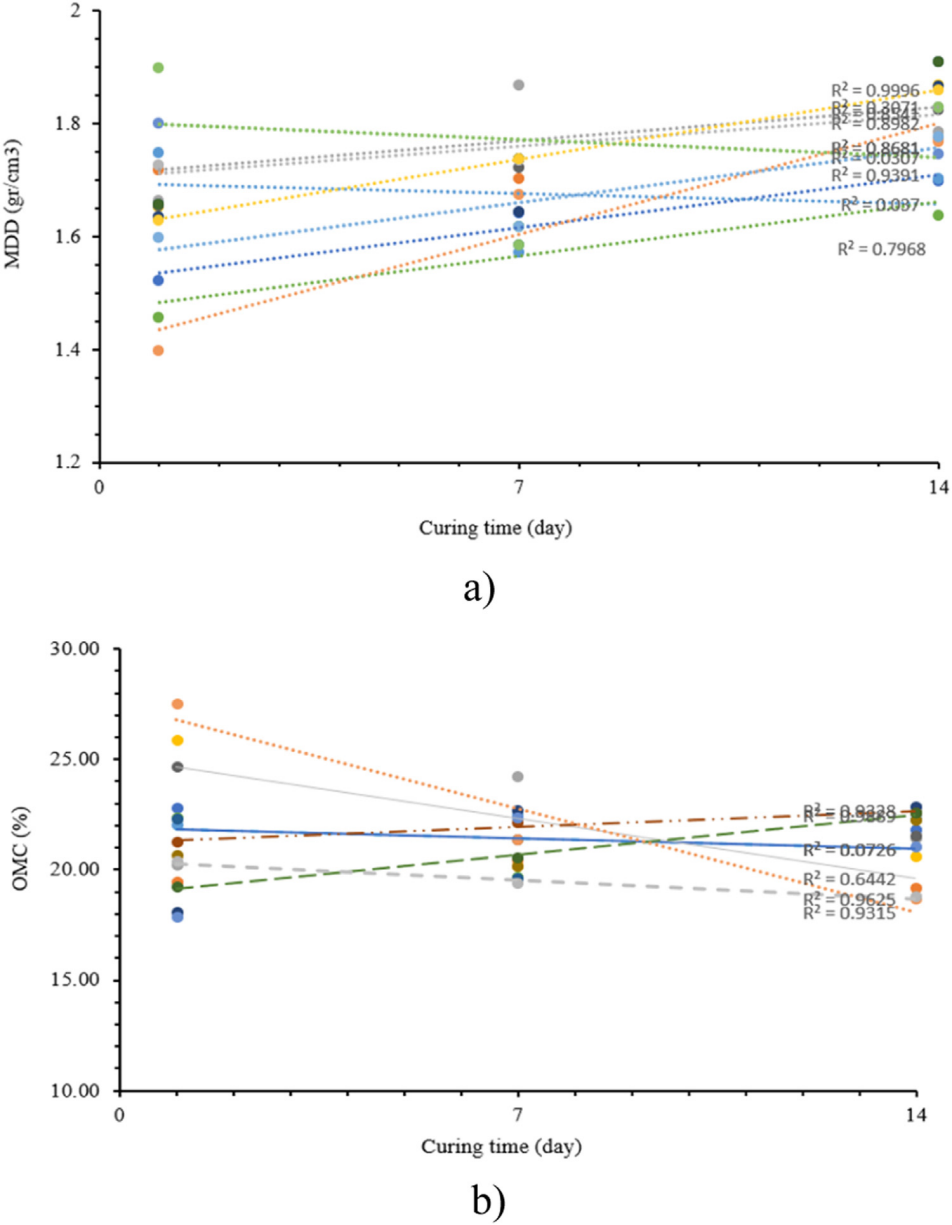
(a) MDD versus curing time (b) OMC versus curing time.

Table 15 shows the three test specimens used to plant in indigenous soils, both with and without stabilization treatment. The table also illustrates the ascending pattern observed in MDD. Subsequently, during the initial day of the planting process, the average MDD of untreated natural soil is observed to be approximately 1.459 gr/cc. However, this value rises to 1.693 gr/cc when soil is subjected to a stabilization treatment consisting of 20 % sand and 5 % lime, shown in Fig. 11.

**Table 15:** MDD value based on curing time.

Mixed composition	Curing time	MDD (gr/cm³)			Average
	(day)	1	2	3	
Original Soil (OS)	1	1.52	1.46	1.40	1.46
	7	1.64	1.62	1.68	1.65
	14	1.70	1.64	1.77	1.70
OS+20 % sand+5 % lime	1	1.72	1.636	1.73	1.69
	7	1.70	1.643	1.74	1.69
	14	1.78	1.865	1.83	1.82
OS+20 % sand+10 % lime	1	1.67	1.66	1.63	1.65
	7	1.87	1.72	1.74	1.78
	14	1.79	1.83	1.86	1.82
OS+20 % sand+15 % lime	1	1.65	1.66	1.60	1.64
	7	1.74	1.739	1.62	1.70
	14	1.87	1.911	1.78	1.85
OS+20 % sand+20 % lime	1	1.75	1.799	1.90	1.82
	7	1.57	1.585	1.59	1.58
	14	1.71	1.745	1.75	1.76

Table 16 shows OMC value for the three test specimens, illustrating a notable decrease in OMC when compared to the initial unstable soil. The average OMC on the initial day of unstable natural soil is recorded at 22.48 %. However, upon stabilization with a mixture consisting of 20 % sand and 5 % lime, OMC decreases to 18.92 % (Fig. 11b).

**Table 16:** OMC value based on curing time.

Mixed composition	Curing time (day)	MDD (gr/cm^3^)			Average
		1	2	3	
Original soil (OS)	1	22.80	22.37	22.28	22.48
	7	19.60	19.65	19.56	19.60
	14	21.78	22.55	20.58	21.64
OS + 20% Sand + 5% Lime	1	19.43	18.09	19.25	18.92
	7	20.47	22.65	20.48	21.20
	14	19.16	22.86	22.58	21.53
OS + 20% Sand + 10% Lime	1	20.25	21.23	17.89	19.79
	7	24.25	22.15	22.36	22.92
	14	21.45	22.56	20.98	21.66
OS + 20% Sand + 15% Lime	1	25.85	24.65	27.54	26.01
	7	20.16	21.37	21.39	20.97
	14	20.61	21.48	18.68	20.26
OS + 20% Sand + 20% Lime	1	21.98	20.65	20.38	21.00
	7	22.45	20.15	19.37	20.66
	14	22.27	22.25	18.78	21.10

## Monitoring and future research suggestions

The design and commissioning of pavement or compaction treatment for future testing are contingent upon the specific purpose of the intended tests. However, it is crucial to comprehend the behavior of MDD and OMC through the examination of various renewable advanced material variations. It is recommended to incorporate a strain meter, transducer, or extensometer in subsequent experiments to effectively monitor different loads, displacements, or strains such methods outlined in [51], [52], [53], [54], [55]. This will provide a more comprehensive understanding of soil specimen deformation, as previously mentioned.

## CRediT authorship contribution statement

**Dandung Novianto:** Conceptualization, Methodology, Writing – review & editing, Supervision, Resources, Validation, Data curation. **Taufiq Rochman:** Resources, Writing – review & editing, Validation, Supervision.

## Declaration of Competing Interest

The authors declare no competing financial interests or personal relationships that could influence the work reported in this study.

## Data Availability

Data will be made available on request.
